# Excessive Use of Salt

**Published:** 1898-07

**Authors:** 


### EXCESSIVE USE OF SALT.

The use of salt as a condiment is so general and so universally believed in as necessary that we rarely hear a word against its excessive use, but there is a multitude of persons who eat far too much salt—eat in on everything, on meat, fish, potatoes, melons, in butter, on tomatoes, turnips and squashes, in bread and on a host of foods too numerous to mention. To so great an extent is it used that no food is relished which has not a salty taste, and this hides more or less the real taste, which is often very delicate. Now, the amount of salt required in the system is comparatively small, and if the diet has been rightly compounded very little is necessary. What are some of the evils of the excessive use of salt? The effect is to paralyze the nerves of taste, or pervert them so they can not enjoy a thing which has not a salty flavor, and in addition there is a direct tax on both the skin and kidneys in removing it from the blood. Whether the skin is harmed by the tax we do not know. Possibly it is not greatly injured, yet we know that few people possess a healthy skin; but it is now pretty well settled that an excessive use of salt does overtax the kidneys in its removal, and that cases of derangement and disease of these organs is due to this use. We advise our readers and others to look into this matter and to try and diminish the excessive use of this condiment. We believe they will be better for it.—*The Clinic.*

